# Acute infective endocarditis masquerading as septic shock with acute respiratory distress syndrome

**DOI:** 10.1002/ccr3.3261

**Published:** 2020-09-26

**Authors:** John W. Davis, Fawad Virk, Masood Ahmad

**Affiliations:** ^1^ Division of Cardiology Department of Internal Medicine University of Texas Medical Branch Galveston TX USA

**Keywords:** echocardiography, mitral valve regurgitation, point‐of‐care screening, sepsis | infective endocarditis

## Abstract

Any new pulmonary edema warrants an urgent rule out of acute cardiac dysfunction. Because sepsis protocols emphasize rapid stabilization and treatment over etiologic evaluation, some cases of endocarditis may be missed as sepsis with acute respiratory distress syndrome.

## Graphical abstract

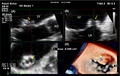

## 1 INTRODUCTION

With increasing emphasis on rapid diagnosis and treatment of sepsis, clinicians may overlook cardiogenic causes of shock. In this report, we discuss a case of acute infective endocarditis that was initially perceived as sepsis complicated by noncardiogenic pulmonary edema, which may be common given emphasis on rapid diagnosis of sepsis today.

Emphasis on rapid sepsis identification and treatment in the emergency department (ED) and intensive care units may lead to missing critical organ involvement underlying the presentation of patients with septic shock. Acute respiratory distress syndrome (ARDS) is one of the sequelae of sepsis, but it is a diagnosis of exclusion and implies noncardiac pulmonary edema. ARDS should not be diagnosed without ruling out cardiac causes, which should at least include an urgent bedside echocardiogram.

## 2 HISTORY OF PRESENTATION

**Figure 1:**
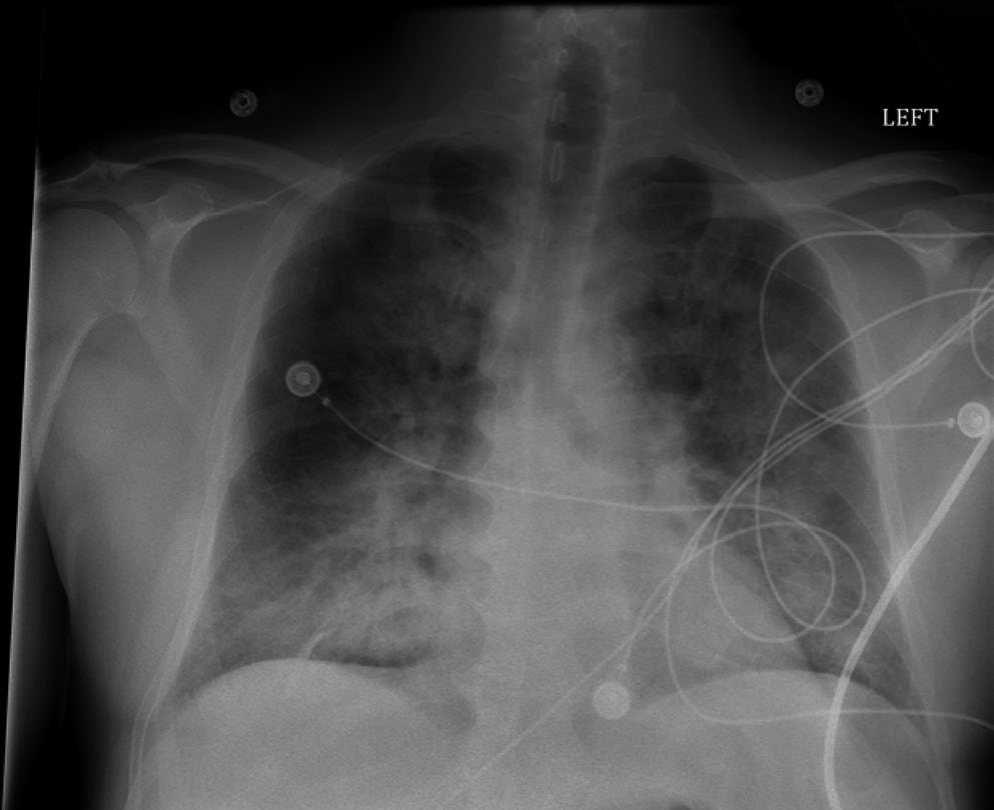
Chest X‐ray PA view, demonstrating acute, bilateral, fluffy pulmonary infiltrates suspected to represent acute respiratory distress syndrome

The patient is a previously healthy 55‐year‐old male who presented to an outside ED with complaints of intractable vomiting, diarrhea, and shortness of breath for the previous 5 days. The patient presented with sinus tachycardia (131 bpm), fever (39°C), blood pressure (131/63 mm Hg), leukocytosis with neutrophilia (19.67 × 10^3^/μL), elevated procalcitonin (1.84 ng/mL), and bibasilar rales on auscultation. There were no abnormal heart sounds or cardiac murmurs noted on examination. Chest X‐ray showed bilateral, “fluffy” pulmonary infiltrates suspected to represent ARDS (Figure 1). ECG demonstrated diffuse and nonspecific ST and T wave changes. Suspecting septic shock of enteric origin, the attending physician empirically started ceftriaxone, levofloxacin, and metronidazole, and transferred the patient to our hospital.

## 3 MANAGEMENT AND PROGRESSION

**Figure 2:**
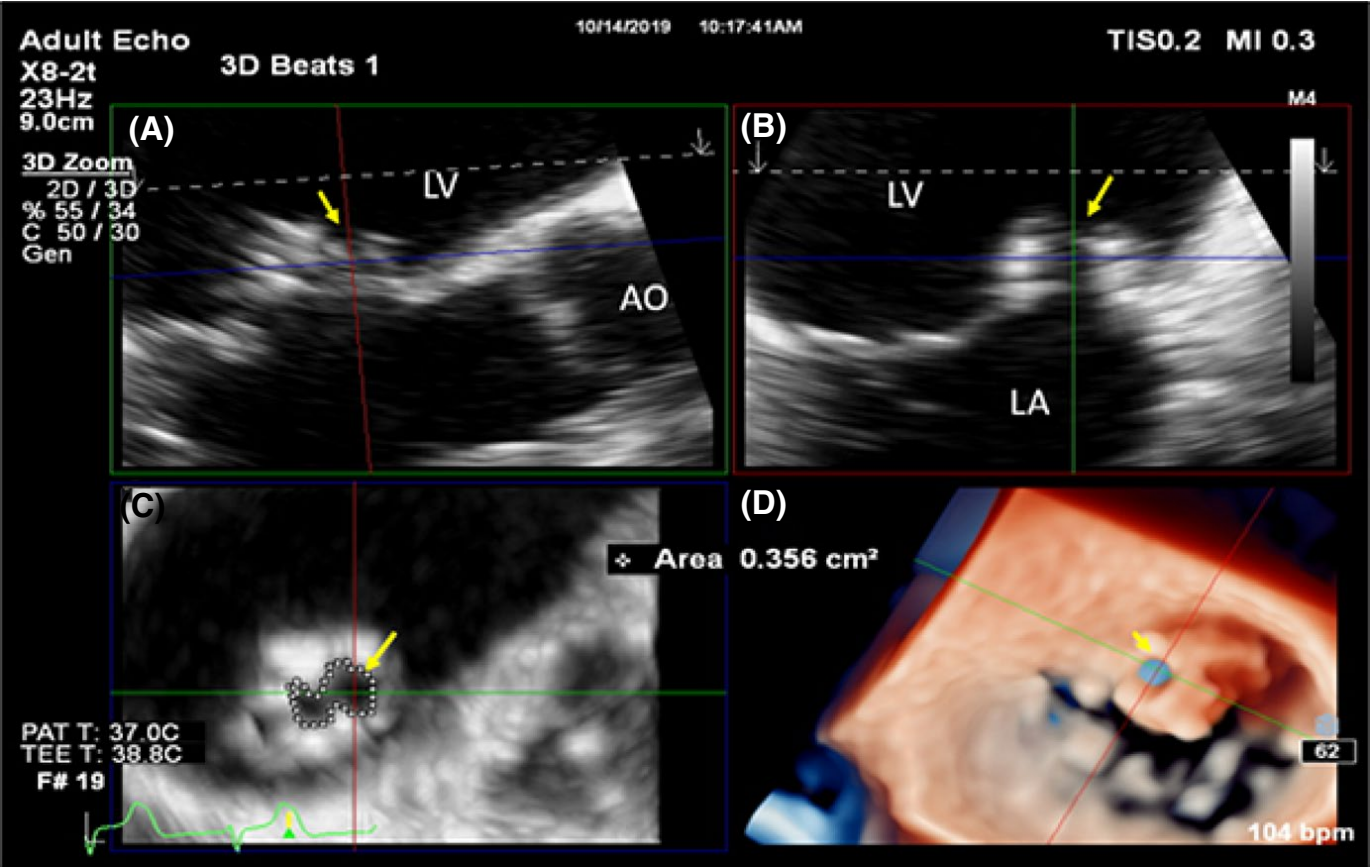
3D TEE multiplanar view of the mitral valve, A (Elevation plane), B (Lateral plane), C (Depth plane), D (3D Volume view)., Arrows point to the vegetation with perforation in the anterior leaflet. AO (Aorta), LA (Left atrium), LV (Left ventricle)

**Figure 3:**
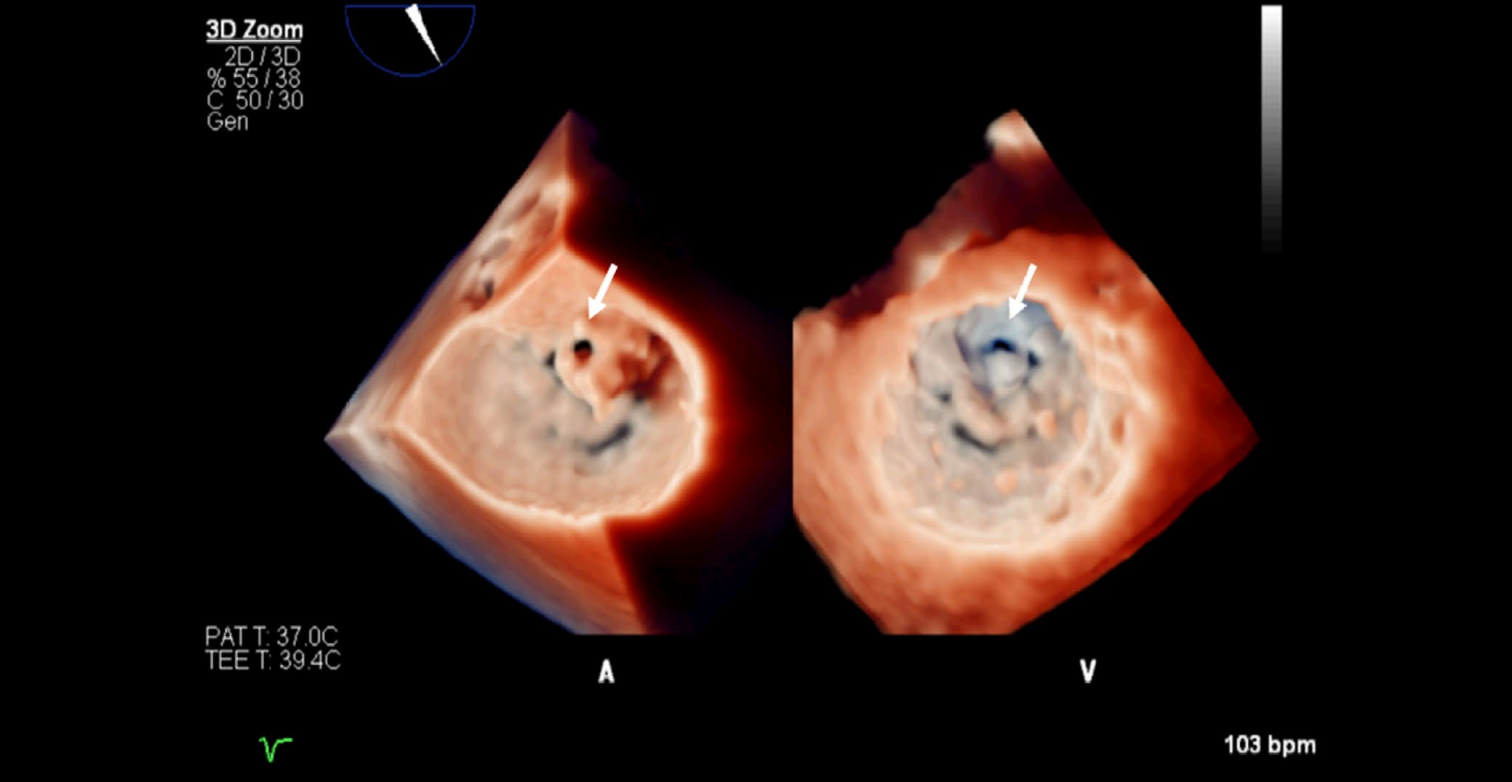
3D TEE True View™ of the mitral valve, Atrial (A) and Ventricular (V) views. Arrows point to the vegetation/perforation in the anterior leaflet

**Figure 4:**
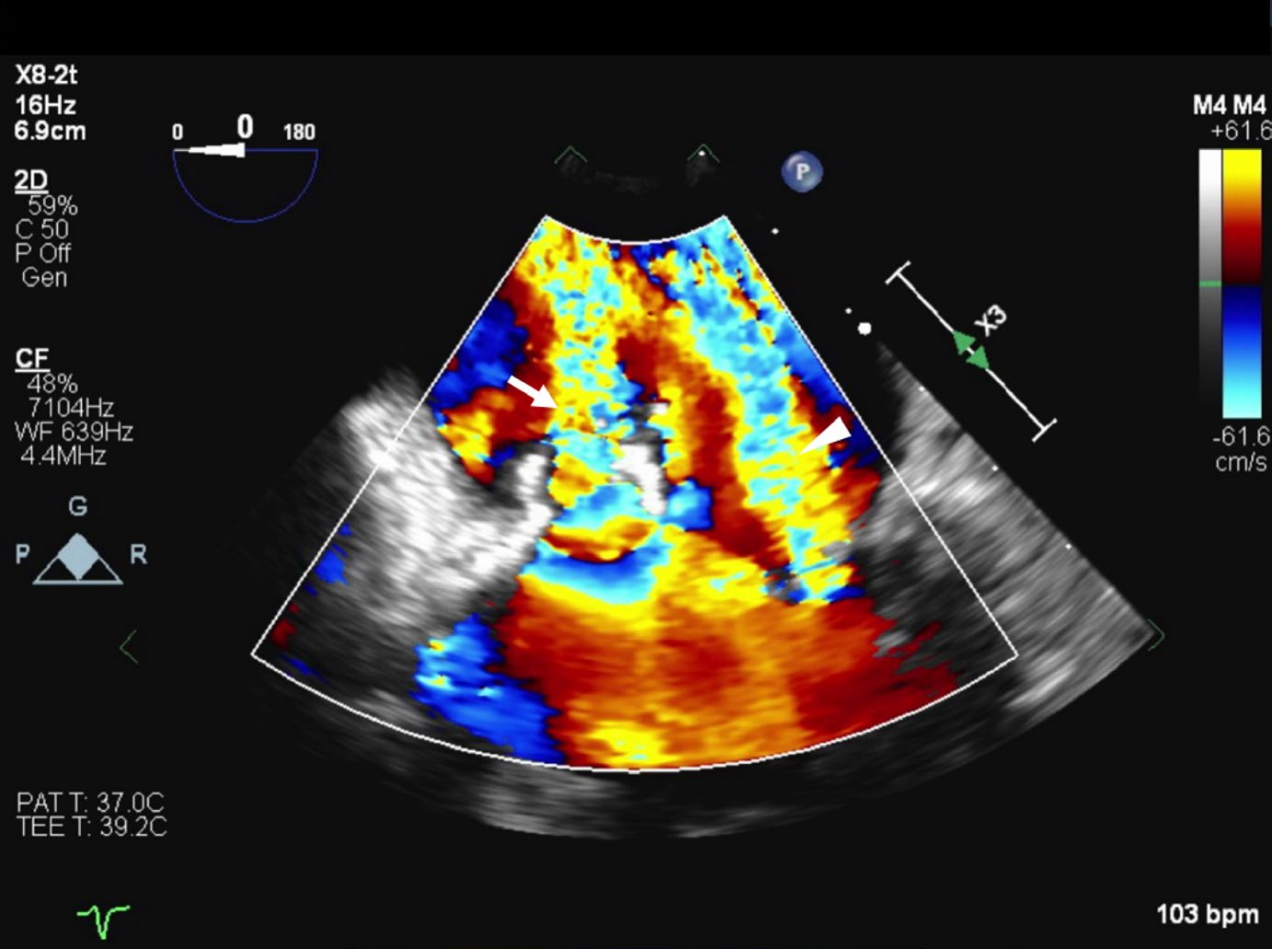
2D transesophageal color flow showing mitral regurgitation through the perforation (arrow) and the mitral orifice (arrowhead)

**Figure 5:**
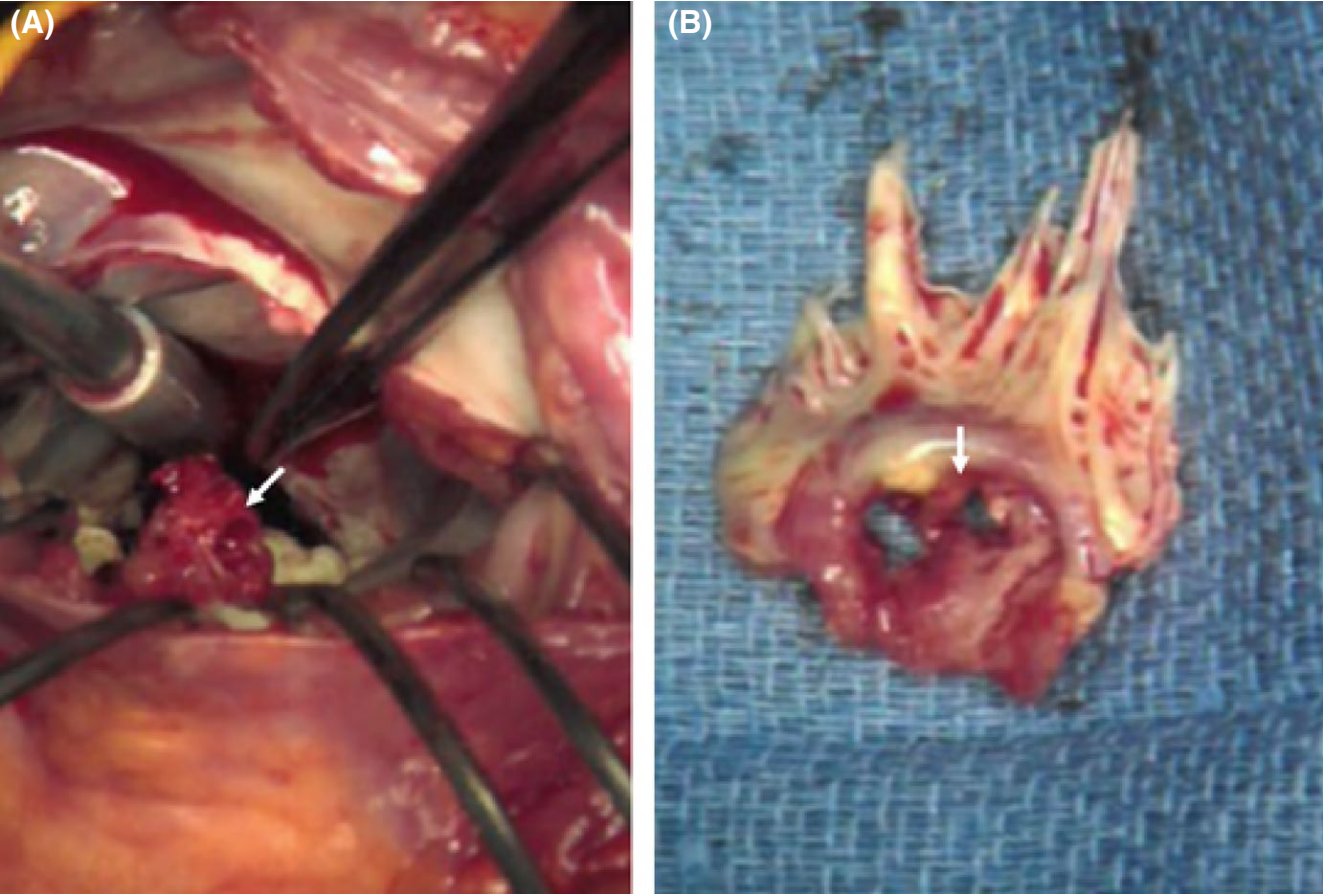
Left atrial (surgical) view of the mitral valve with arrows showing vegetation/perforation in the anterior mitral leaflet. A (Surgical field), B (Excised mitral valve)

Upon arrival at our hospital, the patient's condition deteriorated despite antibiotic therapy. His blood pressure decreased to 86/55 mm Hg, with sinus tachycardia at 115 beats/min. He developed respiratory distress with a respiratory rate of >40/min and arterial PO_2_ of 45 mm Hg, requiring intubation. Following intubation and norepinephrine pressor therapy, the patient's hemodynamic status and oxygen saturation improved. On cardiovascular examination, his jugular venous pressure did not appear elevated. There was no peripheral edema. On auscultation by multiple physicians, there were no gallops or murmurs detected. Cardiac troponins were mildly elevated (0.086 ng/mL, normal < 0.035 ng/mL) and NT‐pro‐BNP was elevated at 1600 pg/mL. The elevation in pro‐BNP and declining O_2_ saturation prompted the attending physician to order urgent bedside echocardiography. Transthoracic echocardiogram was of limited technical quality and showed a mobile mass on the anterior leaflet of the mitral valve (MV) with hyperdynamic left ventricular (LV) systolic function. A subsequent transesophageal echocardiogram revealed a 1.2 × 0.88 cm vegetation attached to the anterior leaflet with a central 1 cm^2^ perforation (Figures 2 and 3, Videos [Link], [Link], [Link]). There was severe mitral regurgitation (MR) through both the valve orifice and the perforation (Figure 4). All blood cultures grew MSSA (methicillin‐sensitive staphylococcus aureus) that was unresponsive to the current antibiotic therapy. The patient was placed on cefazolin treatment and underwent emergent MV replacement. Gross inspection of the valve during surgery confirmed the echocardiographic findings of anterior MV vegetation and perforation (Figure 5). The valve was replaced without complications, and the patient was discharged after one week of continued observation.

## 4 DISCUSSION

Within thirty minutes of the initial presentation, a diagnosis of septic shock was made with presumed enteric etiology. It was later found that a punch biopsy had been performed for psoriasis workup at a dermatology appointment several days prior to presentation. This biopsy became the presumed point of entry for staphylococcal bacteremia. Cardiogenic causes of acute pulmonary edema were not considered until two days into the hospital course, which represents a near miss of a catastrophic outcome. Auscultatory findings, though helpful in identifying most valvular regurgitant flow, may be unremarkable in severe acute MR due to the rapid equilibration of pressures between the left atrium and LV.

Acute MR may be due to structural causes such as leaflet perforation from endocarditis, chordal rupture due to myxomatous degeneration, and papillary muscle rupture in the setting of acute myocardial infarction.^1^ Acute MR may also occur due to nonstructural causes such as functional MR in severe acute ischemia, which often improves with treatment of underlying ischemia. In contrast, acute MR due to structural disruption requires urgent surgical intervention. Any delay in diagnosis may result in an adverse outcome. The diagnosis of severe acute MR at the bedside may be confounded by lack of the usual physical findings present in chronic MR. As a result of acute decrease in cardiac output, high LV diastolic pressure, tachycardia, and preserved left atrial compliance, the systolic murmur of acute MR may be soft and can be brief that is easily missed on physical examination.^1^ In our patient, multiple experienced auscultators were not able to hear a systolic murmur, thus delaying the consideration of acute MR as the cause of pulmonary edema. In the setting of sepsis, the pulmonary process was felt to be noncardiac in origin and the patient was initially treated for presumed ARDS. Thus, reliance on physical examination alone for diagnosis of acute MR may result in delay in urgent care.

In the Third International Consensus report, sepsis is defined as a life‐threatening organ dysfunction caused by a dysregulated host response to infection.^2^ Organ dysfunction can be represented by increase in Sequential Organ Failure Assessment (SOFA) score.^2^ A quick bedside score, quick SOFA (qSOFA) is used for triage of patients who are suspected to have infection and high likelihood of deterioration. The qSOFA score includes respiratory rate of 22/min or greater, altered mental status, or systolic blood pressure of 100 and less.^2^

Our patient was appropriately triaged in the ED and sepsis protocol was started. While sepsis complicated by ARDS is a catastrophic event that leads to significant mortality in Western countries,^3^ the coincidence of ARDS with sepsis is less common than many believe. One study estimated ARDS to be present in less than10% of all sepsis cases.^3^ Given the numerous comorbidities such as congestive heart failure that can present as pulmonary edema in the presence of sepsis, it must be emphasized that any rapid sepsis workup should still include thorough consideration of cardiac involvement.

Infective endocarditis can present with wide a range of symptoms, and it is challenging to make a diagnosis of infective endocarditis in the ED. There are case reports of rapid early diagnosis of infective endocarditis of patients while in the ED using point‐of‐care ultrasound leading to good clinical outcomes.^4^ The mitral valve is often involved in infective endocarditis. In one Italian study, the incidence rate of infective endocarditis was 4.6/100, 000 person years, and 38% of these were mitral valve endocarditis.^5^ The timing of surgery in mitral valve endocarditis is crucial, and early involvement of the surgical team is important. In one randomized study, early surgery (within 48 hours of presentation) for infective endocarditis due to large vegetations showed a significant reduction in all‐cause death and/or systemic embolism compared with conventional therapy.^6^

It has recently been suggested that point‐of‐care echocardiography should become a routine part of rapid sepsis workup in emergency protocols,^7^, ^8^ which would have significantly decreased the time to diagnosis in this patient. This step would have allowed for presumptive diagnosis in the ED as opposed to days later during the patient's admission. While this near miss does not make the full case for incorporation of bedside echocardiography into sepsis protocols, new pulmonary edema in sepsis should immediately trigger echocardiography to rule out cardiogenic complications. The early use of echocardiography would also have improved antibiotic therapy selection, as sepsis with cardiac involvement would necessitate more targeted staphylococcal coverage. Use of bedside echocardiography in this patient likely would have triggered earlier transesophageal echocardiography with 3D enhancement in our patient, which ultimately provided exquisite images and confirmation of the diagnosis.^9^, ^10^

## 5 CONCLUSION

Echocardiography plays an important role in evaluating LV function and identifying valvular disease in shock protocols, especially when auscultation is unremarkable. Rapid equilibration of LV and left atrial pressures in severe MR may attenuate the murmur. Identification of acute valvular endocarditis in the setting of sepsis and severe MR presenting with shock was critical in proceeding with surgical intervention with a good outcome. Sepsis protocols, particularly with identified pulmonary edema, should include urgent bedside echocardiography.

## CONFLICT OF INTEREST

No conflict of interest to disclose.

## AUTHOR CONTRIBUTIONS

JD: responsible for all manuscript drafting and submission, along with selection and curation of images. FV: involved in the responsibility of obtaining the surgical images and for this patient's clinical care. MA: involved as supervising investigator, and responsible for oversight and curation of overall manuscript drafting and image selection.

## ETHICAL APPROVAL

The patient gave verbal consent for his case's representation in a published case report, given the emergent and rapid nature of his clinical course. There were no funders for this research, and the authors deny any conflict of interest that may have influenced the writing of this case report.

## Supporting information

Video S1

Video S2

Video S3
